# An Assay for Systematically Quantifying the Vestibulo-Ocular Reflex to Assess Vestibular Function in Zebrafish Larvae

**DOI:** 10.3389/fncel.2018.00257

**Published:** 2018-08-21

**Authors:** Peng Sun, Yingla Zhang, Feng Zhao, Jian-Ping Wu, Sio Hang Pun, Cheng Peng, Meide Du, Mang I. Vai, Dong Liu, Fangyi Chen

**Affiliations:** ^1^State Key Laboratory of Analog and Mixed-Signal VLSI, University of Macau, Taipa, China; ^2^Department of Electrical and Computer Engineering, Faculty of Science and Technology, University of Macau, Taipa, China; ^3^Department of Biomedical Engineering, Southern University of Science and Technology, Shenzhen, China; ^4^School of Life Sciences, Peking University, Beijing, China; ^5^Department of Biology, Southern University of Science and Technology, Shenzhen, China; ^6^SUSTech Academy for Advanced Interdisciplinary Studies, Southern University of Science and Technology, Shenzhen, China

**Keywords:** vestibulo-ocular reflex, zebrafish larva, otolith, vestibular function, gene knock down

## Abstract

Zebrafish (*Danio rerio*) larvae are widely used to study otic functions because they possess all five typical vertebrate senses including hearing and balance. Powerful genetic tools and the transparent body of the embryo and larva also make zebrafish a unique vertebrate model to study otic development. Due to its small larval size and moisture requirement during experiments, accurately acquiring the vestibulo-ocular reflex (VOR) of zebrafish larva is challenging. In this report, a new VOR testing device has been developed for quantifying linear VOR (LVOR) in zebrafish larva, evoked by the head motion about the earth horizontal axis. The system has a newly designed larva-shaped chamber, by which live fish can be steadily held without anesthesia, and the system is more compact and easier to use than its predecessors. To demonstrate the efficacy of the system, the LVORs in wild-type (WT), *dlx3b* and *dlx4b* morphant zebrafish larvae were measured and the results showed that LVOR amplitudes were consistent with the morphological changes of otoliths induced by morpholino oligonucleotides (MO). Our study represents an important advance to obtain VOR and predict the vestibular conditions in zebrafish.

## Introduction

Vestibular dysfunction, due to aging, genetic diseases, injury, or medical conditions is common today, but a direct measurement or observation of inner ear defects is not feasible. Alternatively, the vestibulo-ocular reflex (VOR) of the eyes, which refers to a reflex caused by tilting the patient's head, has been conventionally used in the clinics for assessment of vestibular function. To compensate the head movement that activates the three-neuron circuits in the vestibular system, our eyes intrinsically rotate in the opposite direction for stabilizing our gaze on the retina and maintaining spatial orientation (Barr et al., 1976; O'leary and Davis, 1990; Dai et al., 2014; Carson et al., 2017). The VOR testing has been successfully adopted in primates (Miles and Fuller, 1974; Merfeld, 1995) and rodents (Curthoys, 1979; Pettorossi et al., 1986).

Zebrafish (*Danio rerio*) has been popularly used to study the inner ear, in which both auditory and vestibular end-organs are physically situated. The zebrafish displays 71.4% homology to human genome (Howe et al., 2013), and ~50 hearing-related zebrafish genes have been identified and many of them function similarly in comparison with other vertebrates (Nicolson, 2005; Howe et al., 2013). Zebrafish has auditory saccule and vestibular utricle in each inner ear to detect acoustic vibrations and linear acceleration, respectively. However, similar to mammalian otocysts, sensory hair cells (HCs) of zebrafish larval membranous labyrinth serve as the receptors for the sound and motion signals. Because zebrafish larvae are a few millimeters in length and live in water, methods to evaluate the vestibular function in human or adult zebrafish are not applicable to assess the vestibular function in fish larva (Kenyon et al., 1998; Trapani and Nicolson, 2010). To assess inner ear functions in zebrafish larvae is largely relied on the behavioral observation and evaluation (Mo et al., 2010; Cameron et al., 2013). However, it becomes clear that C-startle response of free-swimming zebrafish, which has been measured in many previous studies, is a mixed value produced by hearing and noise generated from the swimming (Bang et al., 2002; Neo et al., 2015; Wang et al., 2017; Yang et al., 2017). The unconditioned orienting response of zebrafish larvae to the water currents is used to assess hearing function, yet the visual, vestibular, hydro-mechanical, and proprioceptive cues are all known interfering factors (Platto et al., 2014; Niihori et al., 2015).

Both visual and vestibular senses evoke smooth compensatory eye movements. The visually evoked eye movement is the optokinetic response (OKR) while the vestibular sense-triggered eye movement is named as the VOR. Using a simple and manually driven rotary platform, angular VOR (AVOR) in zebrafish larva sensed by the semicircular canals occurred about 1 h later than the occurrence of OKR in 3 days post fertilization (dpf) (Easter and Nicola, 1997). Mounting zebrafish larva on an auto-rotational and heated (to keep 28°C) stage could be recorded by an infrared (IR) trans-illumination video system in a light-tight box. However, AVOR was undetectable until 35 dpf (Beck et al., 2004). Beck et al. suggested that the AVOR reported in Easter and Nicola (1997) could be partially attributed to the OKR as the visual cues might not be adequately blocked. These attempts to live-monitor VORs have provided very useful information to understand how zebrafish develop vestibular senses. When placed on a stage in a head-down position toward the earth in a dark box, the compensatory eye movements of zebrafish larvae could not be evoked by the semicircular canals or vision. Then, in the 3 dpf larvae, a robust linear VOR (LVOR) response was measured using an IR trans-illumination video system, and the LVOR was relied on sensing a motion input by the otolith organ (Mo et al., 2010). In these previous system, the larvae had to be anesthetized before being glued on a coverslip. Although the mounted larvae are awake during the test, it is hard to estimate how anesthesia affects the eye motion. In addition, the glue and water drop on the head region form a semispherical surface, which causes imaging distortion, or even worse, the shake of culture media drop placed on each larval head could potentially be counted as eye motion responses.

To develop a more accurate device to measure VOR in zebrafish larvae, a specially designed larva-shaped chamber was used to fix zebrafish larvae without using anesthesia. The chamber has been structured to allow better survival of larvae with culture media, and a glass coverslip to prevent semispherical surface and any unwanted wobbling. The efficacy of the assay for the evaluation of the vestibular functions in zebrafish larvae was verified by systematically quantifying the VORs in wild-type (WT) and morphant (Morpholino anti-sense oligo injection at 1–2 cell stage) zebrafish larvae.

## Methods and materials

### Specimens

Wild-type zebrafish (Tübingen strain, TU) were raised and maintained under standardized conditions (Westerfield, 2007). One- and two-cell-stage WT embryos in a single batch were injected with translation-blocking Morpholino oligonucleotides (MO; Gene Tools Inc, Philomath, US) to knock down *dlx3b* and *dlx4b* genes that were shown to regulate the otolith formation and otic HC number (Solomon and Fritz, 2002). Injected and un-injected embryos were raised in ×0.5 E2 embryo media, supplied with 0.002% of methylene blue as a fungicide and fed with dry food (Zeigler Bros Inc., PA, USA) starting at 5 dpf. The specimens used in the current study were 6 dpf larvae, which have both utricular and saccular otoliths in an otic vesicle (OV) with normal development of the inner ear (Riley and Moorman, 2000; Inoue et al., 2013). The otoliths are in fine contact with HCs to initiate auditory or vestibular circuitry signals by deflecting the kinocilia/stereocilia bundles (Shotwell et al., 1981; Inoue et al., 2013). The use of animals was approved by the Ethical Committee of Animal Experimentation of Southern University of Science and Technology.

### Construction of a VOR testing device

**Figure 1:**
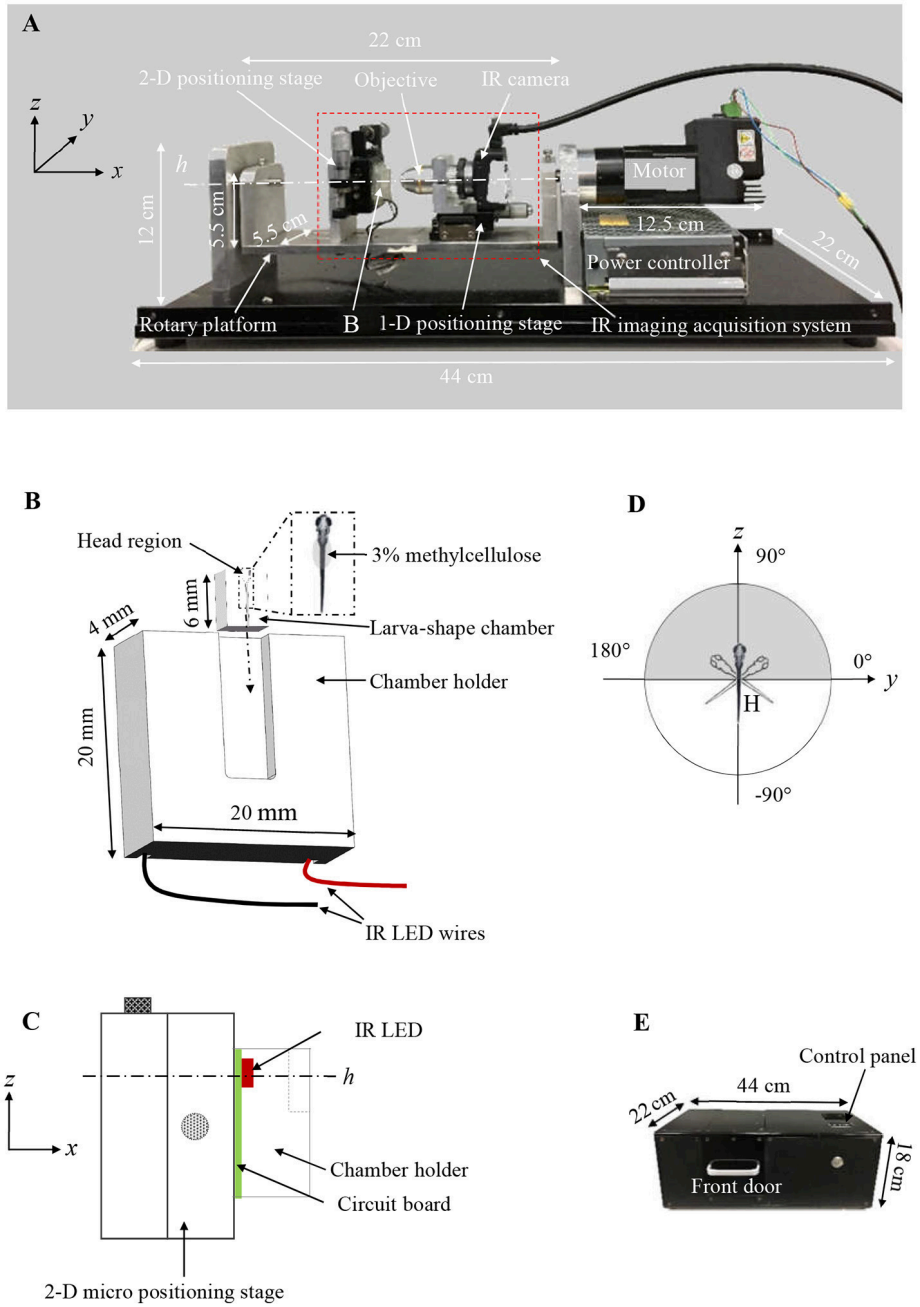
**(A)** Customized VOR testing system. **(B)** A schematic drawing of the larva-shaped chamber and its holder. In the enlarged larva-shaped chamber (dashed rectangle), a larva is embedded in the chamber with 3% methylcellulose, dorsum is facing readers. After the larva-shaped chamber is inserted into its holder (following the dashed arrow), the chamber unit is in a position in which the larval head is up, perpendicular to the rotary platform swinging around the center (*h*) **(C)**, allowing the larva to rotate or “tick-tock” about the earth horizontal axis within its frontal plane (the gray area in **D**). **(C)** A schematic drawing (in the *yz* plane) shows the assembly of the chamber holder **(B)**, IR LED (red) & circuit board (green), the 2-D positioning stage for the *y* & *z* direction. **(D)** A schematic diagram shows the rotatory trajectory of the larva (head) (gray) and the platform (white). **(E)** The black box to house the entire VOR acquisition system.

A new VOR testing device was constructed to measure the eye movements of zebrafish larvae induced by motion (Figure 1A), and its main innovation/functional domains include a larva-shaped chamber (Figure 1B), an IR imaging video system (red dashed box in Figure 1A) for recording stimulated eye responses, and a motorized rotary platform for generating the motion stimuli to the vestibular system. The chamber (6.5 mm × 5 mm × 4 mm, length × width × height) can be inserted into a 5 mm wide slot on a chamber holder. The chamber unit, including its holder, was made of transparent acrylics for trans-illumination. In the chamber, a trench was created to hold the larvae. As shown in Figure 1B, it has a horn-shaped head region and a thin tail region, so that the zebrafish larva can be nicely fitted in. Three percent methylcellulose was applied over the tail for holding the fish in place (Figure 1B). Furthermore, the chamber was specifically structured to leave a space on its top for applying coverslip which not only keeps moisture but also prevents the wobbling and semispherical surface effect.

The chamber unit was fixed on a two-dimensional (2-D) micro positioning stage (Figure 1C) bolted on a rotary platform. An IR LED lamp, installed underneath the chamber unit (Figure 1C), emitted 850 nm IR trans-illumination light for the monochrome IR camera to acquire the eye movements of fish to be tested. A × 10 objective was connected to the monochrome IR camera (Point Gray, Richmond, Canada) that captured at 30-frame/second (fps) with 1024 × 1280 pixels resolution. The monochrome IR camera is fixed on a one-dimensional (1-D) micro positioning stage, which was bolted on the rotary platform. The specimen's eye movements, evoked by the vestibular sense due to the rotational motion, could be captured by the IR camera (Figure 1A). During a typical testing, the larva was in a head-up position, relative to the rotary platform, and rotates about the earth horizontal axis (an area shaded in gray in Figure 1D). Since the chamber and the IR imaging system are fixed on the rotary platform together, there is no relative motion between the larva and the camera during the rotation process, and the eye movement induced only by vestibular stimulation is recorded.

The rotary platform was driven by a stepper motor (model TSM17Q-3AG, MOONS', Shanghai, China) running in a sinusoidal profile. The speed and amplitude of the stepper motor are adjustable via an STM8 microcontroller (STMicroelectronics, Geneva, Switzerland). A gear head (model 42PG1200-010, SAMSR MOTOR, Shanghai, China) with a gear ratio of 1:10 was attached to the stepper motor to convert/reduce the output speed and increase the motor torque. The 2-D and 1-D micro positioning stage were used to adjust the field of the view (FOV) and focus, respectively.

### Data acquisition

**Figure 2:**
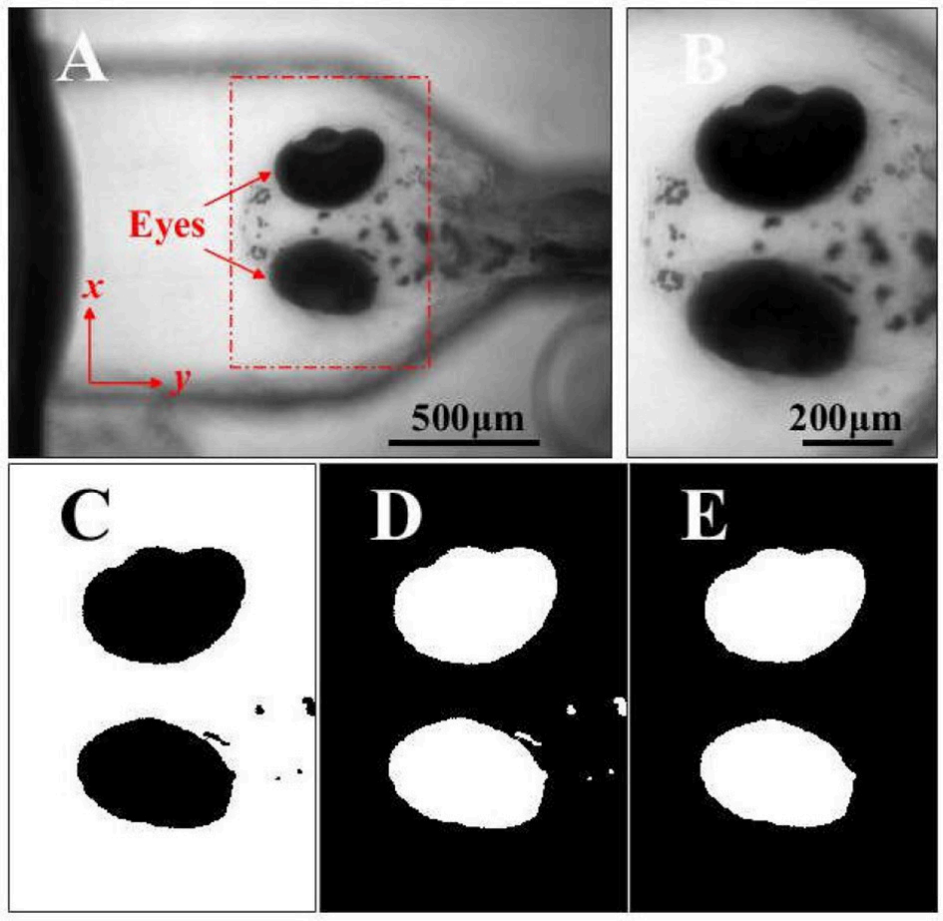
**(A)** The head and eyes (red arrows) of a zebrafish larva acquired in a video series by the VOR test system. **(B)** The ROI containing the eyes is selected and used to analyze the eye movements and VOR. **(C)** The binary image of image in **B** (black eyes). **(D)** The inversed binary image of image in **C** (white eyes). **(E)** The inversed binary image in **D**, where the small flecks are removed by applying the area threshold, and the image is subsequently used to analyze the movements of the eyes stimulated by the rotation. Scale bars: in **(A)** 500 μm for **A**; in **B**, 200 μm for **(B–E)**.

**Figure 3:**
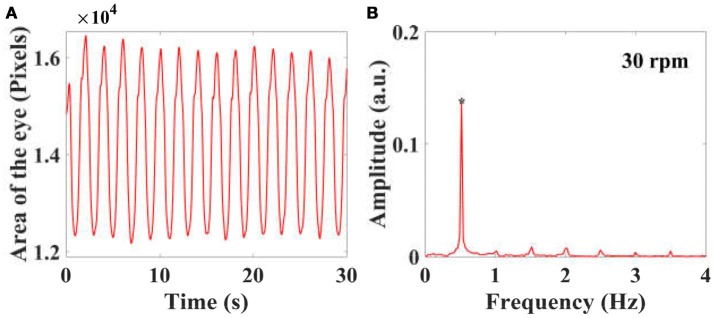
By calculating the projected area of the eyes in a video series in the frontal plane of a larva, the eye movements with time are plotted, as shown in **(A)**. The eye movements in the time domain are then transferred into the spectral domain **(B)** using FFT, and the peaks at stimulus frequency is used to quantify the amplitude of eye movements and VOR.

**Figure 4:**
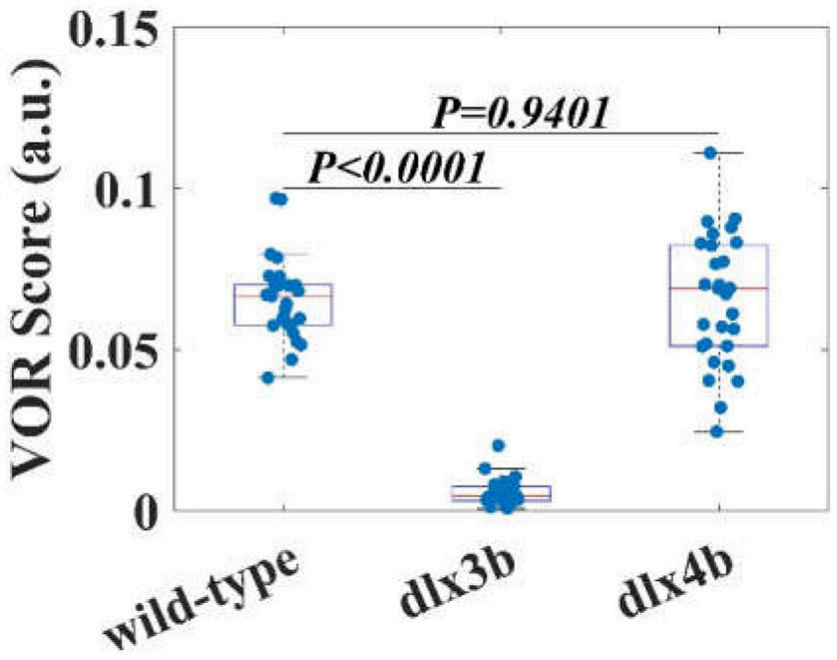
The VOR scores of WT larvae, *dlx3b*, and *dlx4b* morphants. The averaged VOR score of WT larvae is significantly higher than that of *dlx 3b* morphants without the utricular otolith (Figure 6). Although the averaged VOR score of WT and *dlx4b* morphant larvae is not statistically different, all VOR score data points *dlx4b* morphants are more variable.

The IR imaging system recorded series of larval eye movements stimulated by rotation about the earth horizontal axis, and the video recordings were used for quantifying the VOR using a customized computer imaging program (Figures 2–**4**). In one rotation cycle, the platform swung about the earth horizontal axis in a sinusoidal profile (the white part in Figure 1D). The microcontroller automatically sent out a 16-ms pulse to turn off the IR LED for recording one dark frame in the video to indicate the end of a cycle. These dark frames indicate the numbers and finishing position of cycles for full period sampling, which also refer to the highest position of the platform. These dark frames are also used to check the phase lag of eye movements relative to the rotating platform. Up to 1,200 frames of video recording (about 40 s) will be automatically saved as an AVI file and then stored in hard drive connected to the VOR testing system for the subsequent image processing. Video 1 reveals the elaborate steps to acquire VOR. The entire measurement of VOR response was performed in a black box (44 cm × 22 cm × 18 cm, Figure 1E), to ensures that acquired VOR does not include the sensation evoked by visual senses (e.g., OKR).

### Quantification the eye motion

A customized imaging analysis program/software (Supplemental Files), written in MATLAB (Mathworks, Natick, USA), was used to quantitatively analyze the eye movements recorded in a video using the VOR and OKR test device.

#### Quantification of VOR

Figure 2A showed an image of the larval head in the larval frontal plane. The region containing the larval eyes was selected as the region of interest (ROI) in the video frames for quantitatively analyzing the eye movements, as shown in Figure 2A (the red dashed rectangle) and Figure 2B. Then the image of ROI (Figure 2B) would be converted into a binary image (Figure 2C) using Otsu's method (Otsu, 1979). Then the binary image was performed a logical negation where the eyes are white (Figure 2D). the small flecks in the binary image (Figure 2D) were removed through an area threshold and the eyes were extracted for calculating the area of the eyes sequentially in the video (Figure 2E). In this study, a larva was placed in a head-up position perpendicular to the rotary platform, and the larval eyes were found to only rotate approximately about its anterior-posterior (A-P) axis which was not on the image plane. It was not practical to directly measure this rotation using acquired 2-D images. Therefore, we analyzed the projection area of the eyes in the larval frontal plane to quantify the VOR.

To reduce the influence of the eye shape and size of individual larvae in the calculation of the projection area of each eye in the video recording was normalized by the mean value of the eye projection area using the equation: $\bar{X}=\frac{x_{i}-\bar{x}}{\bar{x}}$ where $\bar{x}=\frac{\overset{N}{\underset{i=1}{\sum }}x_{i}}{N}$ is the mean value of the projection area, *x*_*i*_ is the projection area of an eye in the *i*th frame. *N* is the numbers of the frame. After this, the eye change in the time domain were transformed into the spectral domain using Fast-Fourier-Transform (FFT) change, as shown in Figure 3B. In the spectral domain, the peak (^*^ in Figures 3B) at stimulus frequency indicates the amplitude of eye movements or the VOR score of a larva.

#### Quantification OKR

A customized device with a rotating white-and-black striped drum, similar to the one used in Beck et al. (2004) and Scheetz et al. (2018), was constructed to test the OKR. To avoid visual shielding, the aforementioned larva-shaped chamber was modified for OKR test. After being mounted in the modified chamber, larvae were placed on an IR disc-LED which is located in the center of the white-and-black striped drum. Zebrafish eye movements were recorded with an IR camera and the recorded eye image was similar to the one recorded in VOR test. A similar imaging processing method was used to extract the eye. Different from eye rotation in VOR test, the larval eyes rotated about its dorsal-ventral (D-V) axis, which is on the image plane, so that we sequentially calculated the angle between the eye major axis and larval A-P axis. During the test, the drum was rotated back and forth, so that the eye rotation curve is sinusoidal-like and its amplitude or score is calculated for quantifying OKR.

### Experimental procedure

#### VOR testing

To validate the VOR assay developed in this study for assessing the vestibular function in zebrafish larvae, the VOR assay was used to quantify the eye responses of WT, *dlx3b* morphant, and *dlx4b* morphant larvae at 6 dpf to a motion stimulus of 30 rpm.

A zebrafish larva was gently placed in the larva-shaped chamber in a dorsal-up position (Figure 1B; Video 1). The tail of the larva was glued by 3% methylcellulose (Macklin, Shanghai, China) for immobility. After covering a piece of glass coverslip (6.5 cm × 5 cm × 0.17 mm) on the chamber, The head region was filled up with × 0.5 E2 embryo media to support the larval life. The glued larva could freely move its eyes. The chamber unit was then mounted as shown in Figures 1A,C.

After aligning the larval eyes to the center of the FOV of the camera, optimal acquisition was conducted by adjusting the camera shutter, gain and frame rate based on the preview function in the IR camera. The video recording was triggered by pressing the button after the front door of the black box (Figure 1E), and the platform started to rotate at a speed of 30 rpm inducing a vestibular stimulation.

#### OKR testing

To exclude the possibility that MO injection might injure oculomotor neurons and/or afferent visual path, indirectly contributing to vestibular defects if there were any. The OKR of WT and *dlx3b* morphant larvae were measured using the customized OKR device. A zebrafish larva was gently placed in the modified larva-shaped chamber as described in VOR testing. The chamber unit was then placed on the IR disc-LED in a dorsal-up position parallel to the earth. The rotating white-and-black striped drum with spatial frequency of 12.8°/cycle was rotated about the earth vertical axis at the speed of 6 rpm back and forth (± 45°).

#### Microscope observation

To see if the difference in VOR values obtained in above experiments was due to changes of otolith organ (Mo et al., 2010) in each experimental group, the OVs of WT, *dlx3b* morphant, and *dlx4b* morphant larvae were morphologically compared. After the VOR and OKR testing, morphology of the larval ear was captured by a differential interference contrast (DIC) microscope (Zeiss Imager A1, Oberkochen, Germany) for evaluating the correlation of the VOR value and the morphological status of vestibular development.

#### Statistic test

The unpaired two-tailed *t*-test in MATLAB was employed to assess the statistical significance between the independent of data collected from the wild-type vs. morphant larvae in the VOR and OKR test.

## Results

### VOR testing results

Eye responses of WT (*n* = 14), *dlx3b* morphant (*n* = 14), and *dlx4b* morphant (*n* = 15) larvae were shown in Figure 4. Each blue dot in Figure 4 represents a VOR score of zebrafish larval eye. Both WT and *dlx4b* morphant larvae had robust eye movements rotated about the A-P axis. However, the *dlx3b* morphant larva did not show a response although a few larvae showed a response because of defective MO injection (Video 2). As shown in Figure 4, the amplitude of eye movements of VOR in WT larvae (0.0657 ± 0.0128; mean ± standard deviation) was significantly (*p* < 0.0001) higher than that in *dlx3b* morphant (0.0056 ± 0.0042) larvae but indifferent (*p* = 0.9401) from that in *dlx4b* morphant (0.0654 ± 0.0200) larvae. The variation of amplitude of the VOR in WT larvae was smaller than that in *dlx4b* morphant larvae.

### OKR testing results

**Figure 5:**
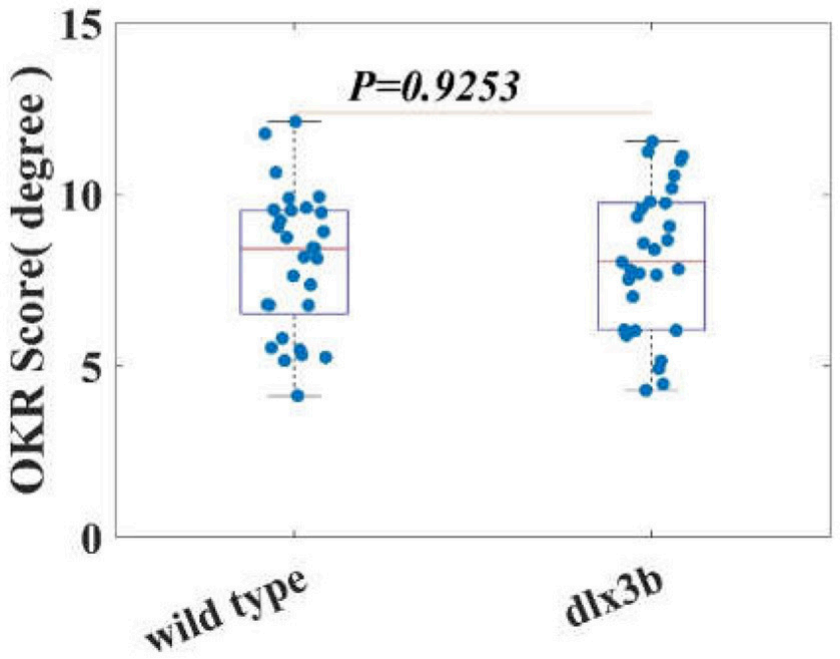
The averaged OKR score of both WT and *dlx3b* morphant larvae are indifferent. indicating a normal oculomotor and visual neural circuit in *dlx3b* morphant larvae.

Angle of OKR in both WT (*n* = 15) and *dlx3b* morphant (*n* = 15) larvae were shown in Figure 5. Each blue dot in Figure 5 represents an OKR score of a zebrafish larval eye. Both WT and *dlx3b* morphant larvae had an evident OKR rotated about the D-V axis (Video 3). As shown in Figure 5, the amplitude of OKR in WT (8.0586 ± 2.0656) and *dlx3b* morphant (8.1104 ± 2.1274) larvae were found to be insignificantly different (*p* = 0.9253), and have similar variations, suggesting a normal visual neural circuit in *dlx3b* morphant larvae.

#### Morphological reduction in larval OV size

**Figure 6:**
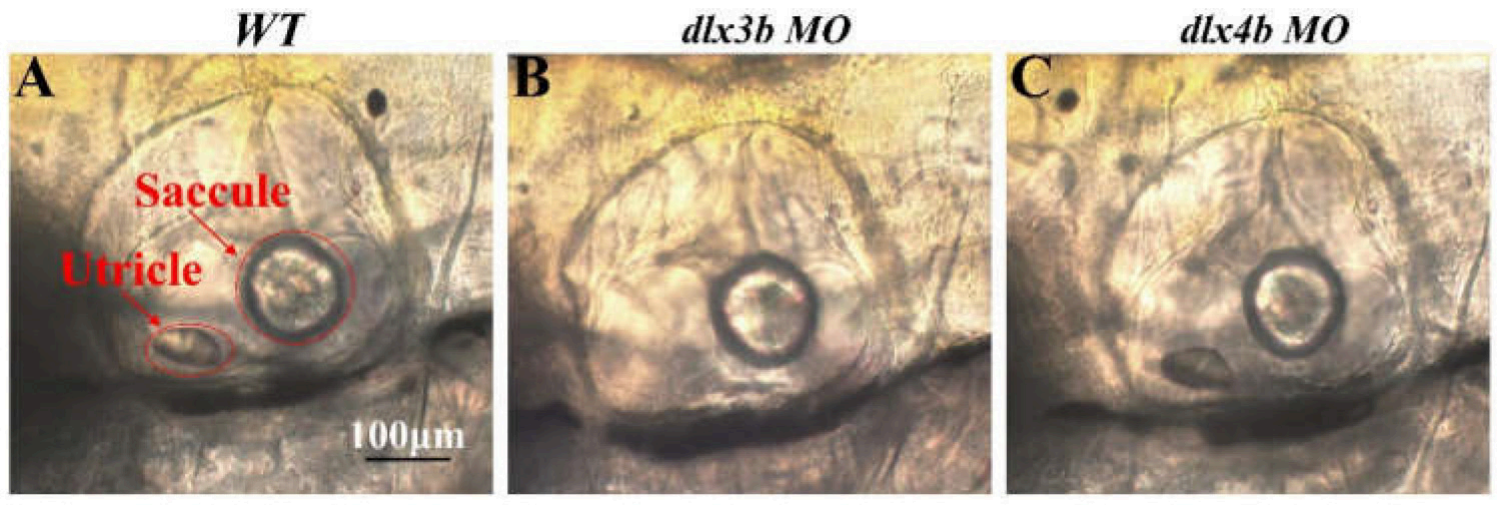
Lateral views, anterior toward the left, of OVs in 6dpf WT, *dlx3b* morphant and *dlx4b* morphant are shown. Most *dlx3b* morphants lacks their utricular otolith, while *dlx4b* morphants retain both otoliths. Scale bars: in **A**, 100 μm for **A–C**.

Positioning anterior toward the left and posterior toward the right in the DIC images, OVs of WT (Figure 6A), *dlx3b* morphant (Figure 6B), and *dlx4b* morphant (Figure 6C) larvae were morphologically compared. OVs of WT and *dlx4b* morphant larvae contained both utricle and saccular otoliths, and the OV of *dlx4b* morphant larva did not display a significant abnormality to that of WT larva. Meanwhile, most of *dlx3b* morphants, which had only the saccular otolith, showed a visible reduction of OV size. These results were consistent to previous observations in 24 or 48 h post fertilization (hpf) embryos (Solomon and Fritz, 2002; Liu et al., 2003).

## Discussion

Although zebrafish does not have a cochlea for sensing sound, the way that the utricle organ senses the motion is very similar to that in humans. Therefore, the VOR testing in zebrafish has provided some useful insights into the cellular mechanisms of the vestibular dysfunction, as well as the correlation between the development of OV and the regulatory genes.

### Am improved VOR testing assay

VOR testing offers a way to assess the vestibular function, so the accuracy of the VOR testing is crucial to not only the assessment of the vestibular function but also understanding the correlation between the VOR score and the vestibular function. In comparing with the previous VOR testing assay (Easter and Nicola, 1997; Beck et al., 2004; Mo et al., 2010), the VOR testing assay developed in this study possesses three main advantages: (1) the VOR testing device (Figure 1A) developed in this study allows acquisition of the VOR in zebrafish larvae without anesthetics; (2) the imaging distortion generated by the semispherical surface of the culture media was completely excluded; (3) the interference induced by the wobbling medium was eliminated. Therefore, the amplitude of VOR acquired using our customized device was closely related to the vestibular function.

### Validation

Since the vestibular function in zebrafish larvae fundamentally relies on the normal development of the vestibular organ, knocking down the regulatory genes that control vestibular organ formation should lead to an abnormal vestibular function (Torres and Giráldez, 1998; Baker and Bronner-Fraser, 2001; Solomon and Fritz, 2002). Previous studies indicated that *dlx3b* and *dlx4b* have redundant function to regulate otolith formation in zebrafish; knocking down *dlx3b*, but not *dlx4b*, led to single-otolith formation in the ear while knocking down/knocking out both genes resulted in no-otolith/small ear vesicle phenotype (Solomon and Fritz, 2002; Liu et al., 2003; Schwarzer et al., 2017). Consistent with the previous studies (Mo et al., 2010; Cameron et al., 2013), the results of our *dlx3b* morphant larvae, which had only saccular otolith in their otic vesicles, suggest that utricular otolith is required for zebrafish LVOR. In addition, since there is a close relationship between the VOR and the genes responsible for regulating the structure of the vestibular organs and microstructure of the utricle, the development of the VOR testing assay may potentially offer a way to screen the genes involved in the development of the vestibular organ.

### Limitation

The eye movements of VOR in larvae are complex and three-dimensional (3-D). In this study, we only analyzed the projection area of the eyes in the frontal plane of the larva to quantify the VOR. We did not find a remarkable difference in the amplitude of VOR between the WT and *dlx4b* morphant larvae. More parameters for characterizing the eye movement in 3-D should be introduced in the future study and, as a result, more comprehensive analysis generating extra information might be performed.

## Conclusions

We have developed a new VOR testing assay to successfully distinguish morphologically abnormal OVs from controls quantifying the amplitude of VOR in zebrafish larvae. Due to the high efficiency of our newly developed VOR testing device, such an assay is promising in screening drugs and mutants that are related to the abnormal vestibular development and/or function.

## Author contributions

FC, PS, and MV conceived and developed the idea. PS, YZ, FZ, and MD conducted Morpholino and VOR experiments. FC, PS, DL, and YZ analyzed the experiment data. FC, DL, PS, and YZ wrote the manuscript. J-PW (ORCID 000-003-4725-2398), SP and CP contributed to the analysis of the data and critical revision of the manuscript.

### Conflict of interest statement

The authors declare that the research was conducted in the absence of any commercial or financial relationships that could be construed as a potential conflict of interest. The reviewer WZ and the handling editor declared their shared affiliation.
